# Multi-region infectious disease prediction modeling based on spatio-temporal graph neural network and the dynamic model

**DOI:** 10.1371/journal.pcbi.1012738

**Published:** 2025-01-09

**Authors:** Xiaoyi Wang, Zhen Jin

**Affiliations:** 1 Complex Systems Research Center, Shanxi University, Taiyuan, Shanxi, China; 2 Key Laboratory of Complex Systems and Data Science of Ministry of Education, Shanxi University, Taiyuan, Shanxi, China; Pennsylvania State University, UNITED STATES OF AMERICA

## Abstract

Human mobility between different regions is a major factor in large-scale outbreaks of infectious diseases. Deep learning models incorporating infectious disease transmission dynamics for predicting the spread of multi-regional outbreaks due to human mobility have become a hot research topic. In this study, we incorporate the Graph Transformer Neural Network and graph learning mechanisms into a metapopulation SIR model to build a hybrid framework, Metapopulation Graph Transformer Neural Network (M-Graphormer), for high-dimensional parameter estimation and multi-regional epidemic prediction. The framework effectively solves the problem that existing models may lose some hidden spatial dependencies in the data when dealing with the dynamic graph structure of the network due to human mobility. We performed multi-wave infectious disease prediction in multiple regions based on real epidemic data. The results show that the framework is capable of performing high-dimensional parameter estimation and accurately predicting epidemic transmission dynamics in multiple regions even with low data quality. In addition, we retrospectively extrapolate the temporal evolution patterns of contact rate under different interventions implemented in different regions, reflecting the dynamics of intervention intensity and the need for flexibility in adjusting interventions in different regions. To provide early warning of infectious disease transmission, we retrospectively predicted the arrival time of infectious diseases using data from the early stages of outbreaks.

## Author summary

In this study, we developed a new method to predict how infectious diseases spread across multiple regions by considering human movement patterns. Our approach combines advanced graph-based deep learning techniques with a classic disease model, creating a powerful framework called M-Graphormer. This framework helps estimate complex disease parameters and predict epidemic trends even when data is scarce or of low quality. By using real-world data from multiple regions, we show that our model can accurately forecast the spread of infectious diseases and adjust for interventions like social distancing or travel restrictions. Additionally, we use our model to understand how different intervention strategies impact the disease’s progression over time. This research is important because it provides tools for public health authorities to anticipate future outbreaks, make better-informed decisions, and implement timely interventions to control the spread of disease. Our findings have the potential to improve the management of global health crises and offer early warnings of epidemic risks.

## Introduction

Over the past three years, the global pandemic of COVID-19 has attracted widespread attention worldwide. With the rapid development of modern transportation, population mobility is accelerating, and disease pathogens are more likely to spread in different regions, so accurately predicting the spread trend of epidemics has become an important task in epidemic prevention and control. A large number of studies have focused on the impact of human mobility on the spread of infectious diseases [1–6], and exploring multi-regional transmission patterns under human mobility can lead to a better understanding of the dynamics of epidemic transmission and the adoption of appropriate control and preventive measures.

Prediction of the spread of infectious diseases in multiple regions can be roughly divided into: infectious disease dynamics methods [7–17], data-driven methods for deep learning [18–24] and deep neural networks combined with infectious disease dynamics methods [25–30].

Infectious disease dynamics aims to establish a mathematical model that can reflect its changing patterns based on the occurrence, development, and environmental changes of the disease. El et al. [7] established a stochastic multi-regional infectious disease model to study the dynamics of infection when infectious diseases occurs in areas connected by any type of anthropological campaign. Fan et al. [8] established a multi-regional SQEIR model to study the impact of global and local interventions on the spread of infectious diseases. In addition, several studies have modeled different multi-regional dynamics based on COVID-19 and evaluated the performance of the models [9, 10]. The metapopulation model is also widely used in multi-regional infectious disease prediction, Feng et al. [11] derived a deterministic non-closed generic model to describe the propagation of diseases on metapopulation networks. Citron et al. [12] compared the dynamics of infectious disease spread in metapopulation models under different population mobility patterns. Das et al. [13] studied the impact of mobility on the spread of COVID-19 by incorporating limited medical resources, isolation, and suppressive behavior of healthy individuals into a metapopulation model. In addition, there are many studies that have established different metapopulation models to study different types of infectious diseases [14–17]. When predictions are made using dynamical models, the epidemiological parameters included in them are usually kept constant [31], whereas in reality they may vary over time, which may lead to the problem of excessive cumulative error when making short-term or long-term predictions. Additionally, the prediction results often rely on model assumptions, which may not fully cover the complexity and uncertainty of the real situation.

Data-driven deep learning is a nonlinear mathematical tool with powerful learning capabilities and is an effective tool for solving many complex problems and tasks. As a typical time series problem, Doni et al. [18] used recurrent neural networks to apply a deep learning approach based on Long Short Term Memory (LSTM) to study the impact of dengue cases in India. Chandra et al. [19] applied LSTM and its three variants for short term COVID-19 prediction in India. In addition, there are many more works for single region infectious disease prediction [20–22]. As mentioned earlier, the spread of infectious diseases usually involves multiple regions, and some studies model it as a spatio-temporal graph structure, using graph neural networks (GNN) and its variants for prediction. La et al. [23] designed a spatio-temporal graph neural network based on cross-location attention, which combines recurrent neural network (RNN) and GNN to capture spatio-temporal dependencies in data for infectious disease prediction. Kapoor et al. [24] proposed a spatio-temporal graphical neural network that learns the complex dynamics inherent in disease modeling and uses the model to predict COVID-19 daily additions of new cases from fine-grained mobility data. However, these studies have ignored the mechanism of infectious diseases, which may lead to a poor understanding of disease spread and impact, and make the results of the modeling poorly interpretable.

The deep neural network combined with the infectious disease dynamics method aims to make the neural network follow the rules of infectious disease dynamics in the learning process, integrating real-time data and complex infectious disease spreading patterns. Kharazmi et al. [25] embedded the integer-order SIR model, fractional-order SIR model, and delay model into the physical information neural network (PINN), so that the model can identify transient parameters and data-driven fractional difference operators. He et al. [26] embedded a SIR model into PINN to identify intervention intensity during the COVID-19 pandemic. Gao et al. [27] proposed a spatio-temporal graphical attention network for infectious disease prediction, which uses a graphical attention network to capture the spatio-temporal trends of disease dynamics and embedded the SIR model in the loss term to enhance the long term prediction accuracy and interpretability of the results. Wang et al. [28] designed an attention-based dynamic GNN module to capture spatial and temporal disease dynamics and provide epidemiological context for node embedding via a dynamic model. It learns spatio-temporal embedding in the latent space of graph input features and epidemiological context and combines them by using a mutual learning mechanism based on graph-based nonlinear transformations. Cao et al. [29] combined GNN into a metapopulation model to explicitly learn infectious disease parameters and potential infectious disease spread graphs from heterogeneous data end-to-end. Mao et al. [30] combined the hybrid gravity metapopulation model into the spatio-temporal graph attention network to adaptively define interactions between regions and help the model learn the dynamics of infectious disease transmission. Most spatio-temporal graph neural networks use GNN or graph attention networks (GAT) when capturing the spatial dependence of node data. In reality, the graph structure formed by population mobility is constantly changing. GNN aggregates node information by an adjacency matrix or an assignment adjacency matrix, which cannot handle dynamic graph structure and needs to transform the dynamic graph structure into a static graph for processing. GAT aggregates node information by similarity measures between nodes, which does not rely on graph structure at all, although it can deal with dynamic graph problems. This results in the loss of some hidden spatial dependencies in the data when dealing with dynamic maps using both neural networks. Ying et al. [32] proposed a Graph Transformer Neural Network (Graphormer) based on the Transformer [33] architecture, which employs three novel graph structure encoding that can effectively solve the above problems (i.e., it has a great advantage for mining the complex relationships of dynamic graph structures).

The main objective of this study is to solve some of the problems of existing studies as described above and to combine observed data from multiple sources with deep learning and epidemiological modeling so that the neural network follows the rules of infectious disease dynamics during the learning process, integrating real-time data and complex infectious disease transmission patterns. To this end, we incorporate the Graphormer and graph learning mechanisms into the metapopulation SIR model to build a hybrid framework, Metapopulation Graph Transformer Neural Network (M-Graphormer), for high-dimensional parameter estimation and multi-regional epidemic prediction. In addition, we extend the three graph structure encoding included in the Graphormer and use a metapopulation model with migration, birth, and death terms to enable the hybrid framework to take full advantage of exploiting dynamic graph structures and to be more consistent with the transmission patterns of multi-regional infectious diseases. M-Graphormer is unique in its ability to model complex relationships between regions, its sensitivity to spatial and temporal variations, and its effectiveness in handling dynamic graph structures. It not only explicitly learns high-dimensional epidemiological parameters from heterogeneous data from multiple sources in an end-to-end manner and simultaneously predicts the disease transmission status in multiple regions, but also exhibits excellent performance when data quality is low. In addition, we further retrospectively inferred the temporal evolution patterns of contact rate under different interventions implemented in different regions and predicted infectious disease arrival times.

## Materials and methods

This study simultaneously predicts the number of daily new cases in multiple regions. Use $G=[G^{1},G^{2},\ldots ,G^{T}]$ to represent the dynamic spatial network, where $G^{t}=\left(V,E^{t}\right)$ is the graph at timestamp *t* with V denoting the set of *N* nodes and $E^{t}$ being the set of directed weighted edges, respectively. For every pair of connected nodes i,j∈V at timestamp *t*, *w*_*ij*_(*t*) ∈ *R* denotes the weight of the directed edge from source node *i* to target node *j* at timestamp *t*. We use

$$\begin{array}{c} A=\left[A^{1},A^{2},\ldots ,A^{T}\right]\in R^{N\times N\times T}, \end{array}$$ (1)

to denote the weighted adjacency matrix sequence. In particular, a weighted adjacency matrix is constructed from mobility data between nodes. To obtain a sparse graph structure, the mobility data threshold is set to 100. Then the edge weight from source node *i* to target node *j* is:

$$\begin{array}{c} w_{ij}\left(t\right)=\{\begin{array}{c} F_{ji}\left(t\right),\;\;\;\;if\;F_{ji}\left(t\right)\geq 100,\\ 0,\;\;\;\;\;\;\;\;\;\;\;\;\;\;\;\;\;\;otherwise, \end{array}\;\;\;\;t=1,2,\ldots ,T. \end{array}$$ (2)

where *F*_*ji*_(*t*) denotes the mobility data between nodes at timestamp *t*, i.e., the number of people moving from source node *i* to target node *j*. It is worth noting that in this paper we do not use additional edge features and only use mobility data to construct the graph structure. Use $X=\left[X^{1},X^{2},\ldots ,X^{T}\right]\in R^{N\times U\times T}$ to represent the node feature matrix, where *X*^*t*^ is the node feature matrix at timestamp *t* and *U* is the number of node features. The input node features include the daily number of new cases, the daily movement changes, *the day of the week*, and daily risk rate. The daily movement change records the change in the range of movement of people compared to the baseline period. The purpose of using the day of the week as an input node feature is to consider different trends and patterns within a week. In some cases, the spread of disease may be affected by different days of the week. For example, people may be more likely to gather or engage in certain behaviors on weekends, which may affect the spread of the disease. Risk rate is an important variable in the transmission process of infectious diseases, indicating the number of new cases in a susceptible population at a given time, and plays a key role in understanding the dynamics of disease transmission within each region. Using this variable as a node feature helps the model to better capture the spatio-temporal dynamics of disease spread. For infectious disease prediction, the objective is to learn a function *f*(⋅), which uses the weighted adjacency matrix *A*^*t*−*T*_*in*_: *t*^ and the node feature matrices *X*^*t*−*T*_*in*_: *t*^ of historical *T*_*in*_ days as inputs, to predict the number of new cases per day for the next *T*_*out*_ days. Where *t* denotes the timestamp, for example, when t denotes 20 November, *X*^*t*−*T*_*in*_: *t*^ denotes the node feature tensor matrix for 20 November and the *T*_*in*_ days before. This problem can be expressed as follows:

$$\begin{array}{c} \left[X^{t-T_{in}:t},A^{t-T_{in}:t}\right]\overset{f(\cdot )}{\to }Y^{t+1:t+T_{out}}, \end{array}$$ (3)

where $Y^{t+1:t+T_{out}}=\left[Y^{t+1},Y^{t+2},\ldots ,Y^{t+T_{out}}\right]\in R^{N\times T_{out}}$ denotes the prediction of daily new cases for all nodes in the next *T*_*out*_ days. *Y*^*t*+1^ denotes the daily new case prediction for all nodes at timestamp *t* + 1.

**Fig 1:**
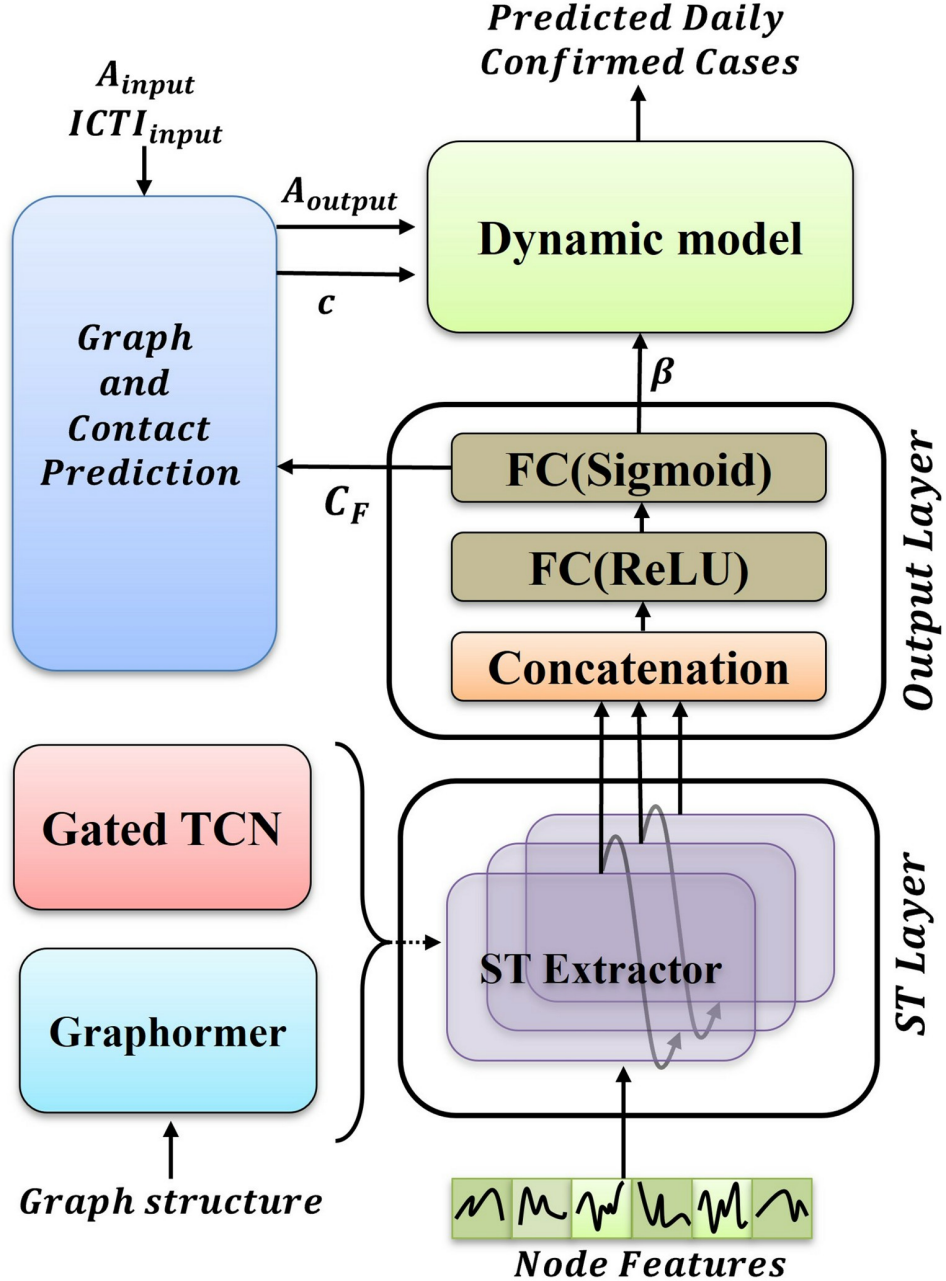
The framework of M-Graphormer. In the output layer, FC represents the fully connected layer, and ReLU and Sigmoid represent the ReLU activation function and Sigmoid activation function respectively.

In this study, to solve the above problem, we built a hybrid framework, Metapopulation Graph Transformer Neural Network (M-Graphormer), for high-dimensional parameter estimation and prediction of infectious diseases, and its overall framework is shown in Fig 1. It consists of three main components: Spatio-Temporal Layer(ST Layer), Graph and Contact Prediction Layer, and Dynamic model. The specific implementation methods of these three parts will be introduced in detail in the following sections.

**Fig 2:**
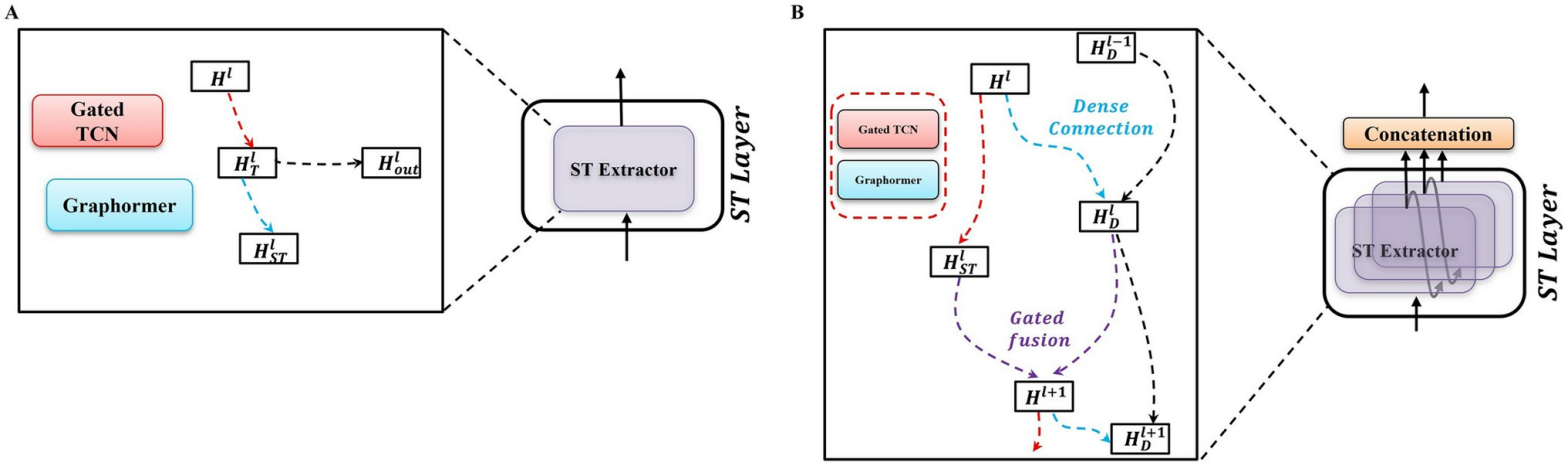
Framework of Spatio-Temporal Layer. A: Framework of Single-layer ST Extractor. B: Framework of Multi-layer ST Extractor.

A single ST Layer contains only one Spatio-Temporal Extractor(ST Extractor), which consists of Graphormer Layer and Gated Temporal Convolution (Gated TCN) Layer, as shown in Fig 2A. The node features $X^{t-T_{in}:t}$ are first input to the Gated TCN to capture the temporal information of the data, and then passed to the Graphormer to capture the spatial information of the data. For convenience, we define the Gated Temporal Convolution Layer and the Graphormer Layer as the Gated TCN operator and the Graphormer operator, as follows:

$$\begin{array}{c} H_{T}^{l}=GatedTCN\left(H^{l}\right), \end{array}$$ (4)

$$\begin{array}{c} H_{ST}^{l}=Graphormer\left(H_{T}^{l}\right), \end{array}$$ (5)

where *H*^*l*^ is the input to the *l*^*th*^ ST Extractor and $H^{l}=X^{t-T_{in}:t}$ when *l* = 0, $H_{T}^{l}$ is the hidden representation containing temporal information, which is used as the input to the Graphormer Layer, and $H_{ST}^{l}$ is the hidden representation containing temporal and spatial information. By stacking multiple ST Layers (i.e. stacking multiple ST Extractors), the M-Graphormer is able to deal with spatial dependencies at different temporal levels, as shown in Fig 2B. The bottom Graphormer receives short-term temporal information and the top Graphormer receives long-term temporal information. Densely connecting different ST Extractors using a gating system, which can extract important information from the previous ST Extractor to pass on to the next ST Extractor:

$$\begin{array}{c} H_{D}^{l}=\{\begin{array}{c} X,\;\;\;\;\;\;\;\;\;\;\;\;\;\;\;\;\;\;\;\;\;\;if\;l=0,\\ H_{D}^{l-1}+H^{l},\;\;\;\;\;\;\;otherwise, \end{array} \end{array}$$ (6)

$$\begin{array}{c} H^{l+1}=H_{ST}^{l}⊙\sigma \left(H_{ST}^{l}\right)+H_{D}^{l}⊙\left(1-\sigma \left(H_{ST}^{l}\right)\right), \end{array}$$ (7)

where $H_{D}^{l}$ is used to store the information from previous layers and *σ* denotes the sigmoid activation function. The core idea of gated dense connections is to connect each layer to all previous layers. This connection method allows information to flow more efficiently in the network, thereby alleviating the gradient vanishing problem. The output of each layer not only depends on the input of the current layer, but also utilizes the features of the previous layer, thereby increasing the reuse rate of features. Then, the outputs of different ST Extractors are connected by skip connections, fusing the information of different scales and obtaining the contact and propagation probability matrix $\beta \in R^{N\times T_{out}}$ for all patches on *T*_*out*_ days through the output layer, and the contact rate influence factor matrix $C_{F}=\left[C_{F}^{1},C_{F}^{2},\ldots ,C_{F}^{T_{out}}\right]\in R^{N\times T_{out}}$, where $C_{F}^{t}=\left[C_{F_{1}}^{t},C_{F_{2}}^{t},\ldots ,C_{F_{n}}^{t}\right]$ represents the contact rate influence factor of all nodes at timestamp *t*. The contact rate influence factor matrix *C*_*F*_ is input into the Graph and Contact Prediction Layer to obtain the contact rate matrix $c\in R^{N\times T_{out}}$ and the weighted adjacency matrix $A^{t+1:t+T_{out}}$, and is input along with *β* into the dynamic model. Using the predicted *β*, *c* and $A^{t+1:t+T_{out}}$, *S*, *I*, *R*, *I*^*cum*^ can be iteratively updated by the dynamic model:

$$\begin{array}{c} \left[S_{n}^{t},I_{n}^{t},R_{n}^{t},I_{n}^{cum^{t}}\right]\to \left[S_{n}^{t+1},I_{n}^{t+1},R_{n}^{t+1},I_{n}^{cum^{t+1}}\right]. \end{array}$$ (8)

We use the Mean Square Error as the loss function of the M-Graphormer, which is defined as:

$$\begin{array}{c} Loss=\frac{1}{NT_{out}}\sum_{i=1}^{N}\sum_{j=1}^{T_{out}}{\left({\hat{I}}_{i}^{cum^{t+j}}-I_{i}^{cum^{t+j}}\right)}^{2}+\frac{1}{NT_{out}}\sum_{i=1}^{N}\sum_{j=1}^{T_{out}}{\left({\hat{I}}_{i}^{new^{t+j}}-I_{i}^{new^{t+j}}\right)}^{2}, \end{array}$$ (9)

where ${\hat{I}}_{i}^{cum^{t+j}}$ and $I_{i}^{cum^{t+j}}$ are the predicted and true cumulative number of confirmed cases at time step *t* + *j* for patch *i*, respectively, and ${\hat{I}}_{i}^{new^{t+j}}$ and $I_{i}^{new^{t+j}}$ are the predicted and true daily number of new cases at time step *t* + *j* for patch *i*, respectively.

### The dynamic model

We extend the model of metapopulation infectious disease dynamics in [34] to simulate multi-regional infectious disease transmission. It consists of any *n* patches (also called sub-population), where individuals within each patch are uniformly mixed and subdivided into three compartments: susceptible, infected, and recovered classes, whose numbers are denoted by *S*_*i*_, *I*_*i*_, *R*_*i*_, where *i* denotes the *i*^*th*^ patch. The total number of individuals is *N*(*t*), and the *i*^*th*^ patch has a total number of individuals of *N*_*i*_(*t*), satisfying

$$\begin{array}{c} N\left(t\right)=\sum_{i=1}^{n}\left(S_{i}\left(t\right)+I_{i}\left(t\right)+R_{i}\left(t\right)\right)=\sum_{i=1}^{n}N_{i}\left(t\right). \end{array}$$ (10)

**Fig 3:**
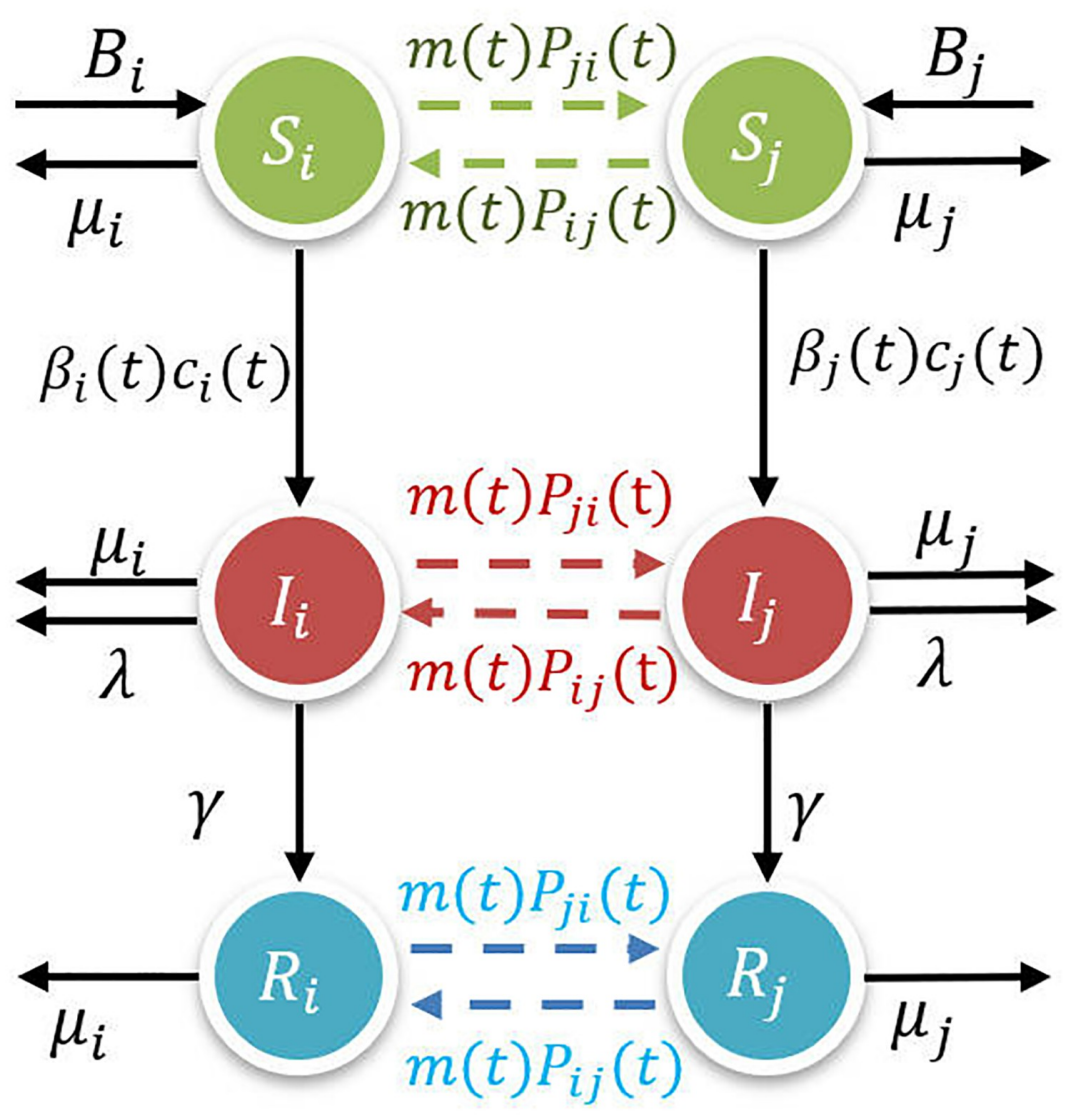
Flow diagram of the metapopulation model (12) showing interactions between patch *i* and patch *j*.

Fig 3 represents the interactions within the two patches and the mobility between patches. All newborns are susceptible and the birth rate is denoted as *B*_*i*_. Because the intervention is constantly adjusted, the contact rate and the probability of contact transmission vary over time and are denoted as *c*_*i*_(*t*) and *β*_*i*_(*t*), respectively. Infected individuals leave the infected compartment with a recovery rate constant *γ* and enter the recovery compartment. Recovered individuals are assumed to be fully immune and will not be reinfected for the length of time considered here. All individuals have natural deaths, and the natural death rate is denoted as *μ*_*i*_, while infected individuals have deaths due to disease and the death rate due to disease is denoted as λ. Individuals are assumed to be mobile between patches by car, train, or plane. Once an individual from patch *i* arrives at patch *j*, that individual mixes evenly with the individuals in patch *j* and is counted as an individual in patch *j*. *F*_*ij*_(*t*) quantifies the number of individuals migrating from patch *j* to patch *i*, and the total number of mobile individuals in the system is

$$\begin{array}{c} \Phi \left(t\right)=\sum_{i=1}^{n}\sum_{j=1}^{n}F_{ij}\left(t\right)\;\;\left(i\neq j\right). \end{array}$$ (11)

Individuals in patch *i* migrate to patch *j* at a rate of *m*(*t*)*P*_*ji*_(*t*), where the average migration rate coefficient $m\left(t\right)=\frac{\Phi (t)}{N(t)}$, $P_{ji}\left(t\right)=\frac{F_{ji}\left(t\right)}{\sum_{j\neq i}F_{ji}\left(t\right)}$ denotes the proportion of individuals migrating from patch *i* to patch *j*. Therefore, we have the following ordinary differential equation:

$$\begin{array}{c} \left\{ \begin{array}{c} \frac{dS_{i}}{dt}=B_{i}-\mu_{i}S_{i}-\frac{S_{i}}{N_{i}}\beta_{i}\left(t\right)c_{i}\left(t\right)I_{i}+m\left(t\right)\sum_{j\neq i}P_{ij}\left(t\right)\left(S_{j}-S_{i}\right),\\ \frac{dI_{i}}{dt}=\frac{S_{i}}{N_{i}}\beta_{i}\left(t\right)c_{i}\left(t\right)I_{i}-\left(\mu_{i}+\lambda +\gamma \right)I_{i}+m\left(t\right)\sum_{j\neq i}P_{ij}\left(t\right)\left(I_{j}-I_{i}\right),\\ \frac{dR_{i}}{dt}=\gamma I_{i}-\mu_{i}R_{i}+m\left(t\right)\sum_{j\neq i}P_{ij}\left(t\right)\left(R_{j}-R_{i}\right). \end{array}\right. \end{array}$$ (12)

The special case of the system, i.e., when births and deaths are ignored and all parameters are constants, is modeled as [34]. Here, consider an additional auxiliary compartment to record cumulative cases, and the dynamics of this compartment are driven by the following equations:

$$\begin{array}{c} \frac{dI_{i}^{cum}}{dt}=\frac{S_{i}}{N_{i}}\beta_{i}\left(t\right)c_{i}\left(t\right)I_{i}. \end{array}$$ (13)

The basic reproduction number *R*_0_ is an important parameter in epidemiology, used to measure the ability of infectious diseases to spread among the population. It defines the average number of individuals that an infected person infects a population of entirely susceptible people without any intervention. The effective reproduction number *R_e_*(*t*) reflects the spread dynamics of the epidemic and the effectiveness of intervention measures. It indicates how many other people each infected person spreads the virus to given the current intervention measures and stage of the epidemic. The effective reproduction number of patch *i* is:

$$\begin{array}{c} R_{e}^{i}\left(t\right)=\frac{\beta_{i}\left(t\right)c_{i}\left(t\right)}{\mu_{i}+\lambda +\gamma +m\left(t\right)}\cdot \frac{S_{i}\left(t\right)}{N_{i}\left(t\right)}. \end{array}$$ (14)

### Graphormer Layer

Transformer is a deep learning model architecture designed for processing sequence data. Its core is a self-attention mechanism. Let $H={\left[h_{1}^{⊤},h_{2}^{⊤},\ldots ,h_{n}^{⊤}\right]}^{⊤}\in R^{n\times d}$ represent the input of the self-attention mechanism, where *d* represents the hidden dimension and *h*_*i*_ ∈ *R*^1×*d*^ is the hidden representation at position *i*. The input *H* is passed through three weight matrices $W_{Q}\in R^{d\times d_{K}},W_{K}\in R^{d\times d_{K}}$ and $W_{V}\in R^{d\times d_{V}}$ to obtain the corresponding representation *Q*, *K*, *V*. Self-attention can be calculated using the following formula:

$$\begin{array}{c} Q=HW_{Q},K=HW_{K},V=HW_{V}, \end{array}$$ (15)

$$\begin{array}{c} \Psi^{attn}=\frac{QK^{⊤}}{\sqrt{d_{K}}},\;Attn\left(H\right)=softmax\left(\Psi^{attn}\right)V, \end{array}$$ (16)

where Ψ^*attn*^ is the similarity matrix capturing the similarity between queries and keys, and *softmax*(⋅) represents the *softmax* activation function. In the Transformer model, the attention distribution is calculated through the semantic correlation between nodes. To incorporate the structural information of the graph into the Transformer model, the Graphormer includes three encoding designs: center encoding, spatial encoding, and edge encoding.

#### Centrality encoding

**Fig 4:**
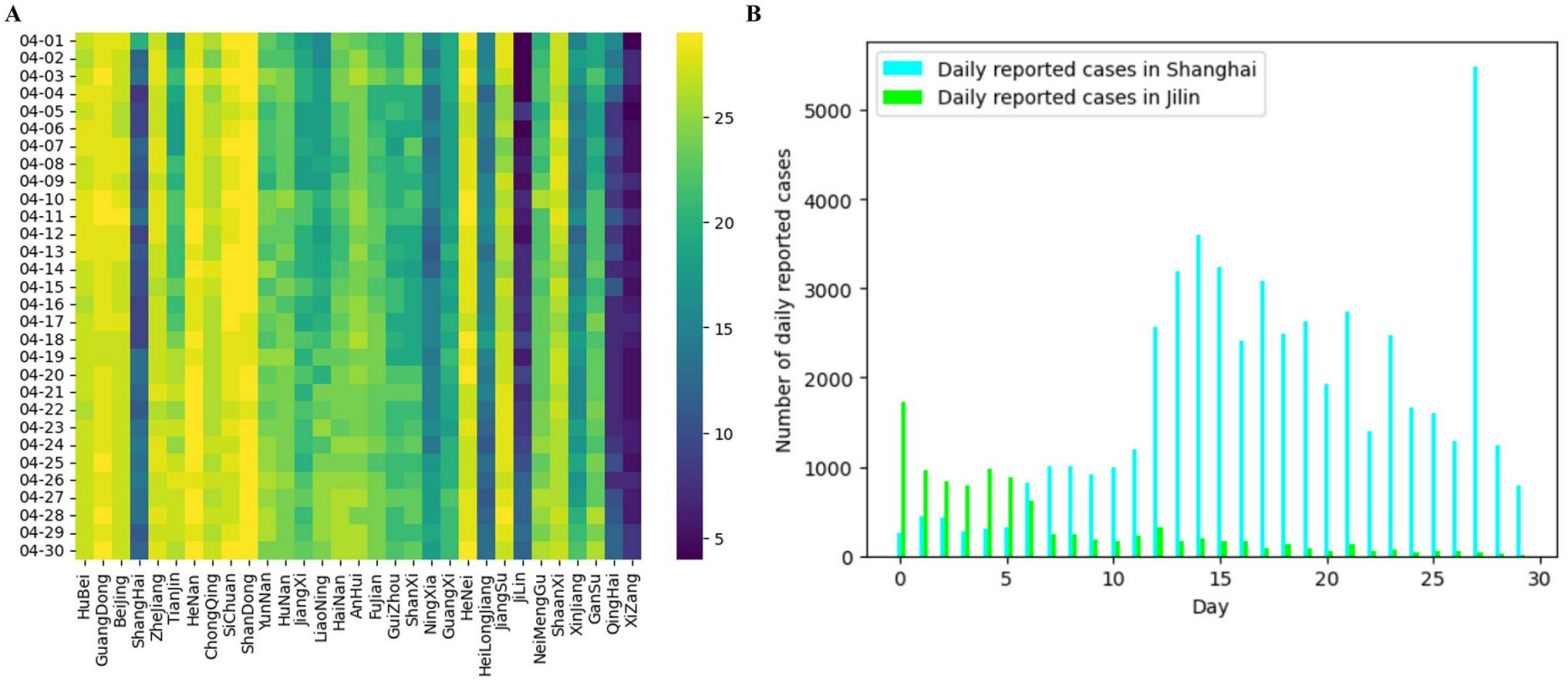
In-degree heat map of Chinese provinces and daily new cases in Jilin and Shanghai in April 2022. A: Heat map of in-degree in April 2022 for each province in China. B: Daily new cases in Shanghai and Jilin, April 2022.

Node centrality is important for measuring the importance of a node in the graph and is, therefore, valuable for attention calculations. To use it as an additional signal for the general network, the Graphormer adopts degree centrality, one of the standard centrality measures. Specifically, each node is assigned two real-valued embedding vectors based on its in-degree and out-degree, which are summed with node features as input:

$$\begin{array}{c} h_{i}^{\left(0\right)}=X_{i}+z_{{\text{deg}}^{-}\left(v_{i}\right)}^{-}+z_{{\text{deg}}^{+}\left(v_{i}\right)}^{+}, \end{array}$$ (17)

where *z*^−^, *z*^+^ ∈ *R*^*d*^ are learnable embedding vectors specified by the in-degree deg^−^(*v*_*i*_) and out-degree deg^+^(*v*_*i*_), respectively. Combining centrality encoding and attention mechanisms, the model is able to consider both semantic relevance and node importance. This helps ensure that the model pays more attention to important nodes during the information dissemination process, thereby improving the model’s understanding and expression of the graph structure. For example, regarding the spread of COVID-19 in China, the country has adopted a dynamic zeroing policy. When COVID-19 spreads in a city, there will be fewer cities connected to it as a result of the policy intervention; i.e., the in-degrees and out-degrees will be smaller than they would have been in the city without the outbreak. As shown in Fig 4, the change in-degree corresponding to the course of the epidemic can be clearly seen through the daily new cases in Jilin and Shanghai during this period. Therefore, the center encoding is promoted to the following form, in which the in-degree and out-degree of the node are used as the two-dimensional features of the node to contact the original features. This can avoid the loss of some hidden features caused by feature summation in Eq (17):

$$\begin{array}{c} h_{i}^{\left(0\right)}=[X_{i}\left|\right|{\text{deg}}^{-}\left(v_{i}\right)\left|\right|{\text{deg}}^{+}\left(v_{i}\right)], \end{array}$$ (18)

where || is the concatenation operator.

#### Spatial encoding

Spatial encoding in the Transformer is used to represent the positional information of labels in a sequence, allowing the model to distinguish between labels at different positions. In graph data, there is no natural order and we are concerned with the neighborhood information of nodes rather than linear sequences. Therefore, positional encoding and locality are handled differently in graphs than in sequences. In order to quantify the spatial relationship between pairs of nodes in the graph and enhance the model’s ability to understand the spatial layout of nodes in the graph, the Graphormer uses a new spatial encoding method. For any graph, the spatial relationship of node pairs in the graph is measured by the function *ϕ*(*v*_*i*_, *v*_*j*_):*V* × *V* → *R*. Assign each output value a learnable scalar and use it as a bias term affecting the Query-Key product matrix Ψ^*attn*^, thus defining the *i*^*th*^ row and *j*^*th*^ column elements of the matrix $\Psi_{ij}^{attn}$ as:

$$\begin{array}{c} \Psi_{ij}^{attn}=\frac{\left(h_{i}W_{Q}\right){\left(h_{j}W_{K}\right)}^{⊤}}{\sqrt{d}}+b_{\phi (v_{i},v_{j})}, \end{array}$$ (19)

where *b*_*ϕ*(*v*_*i*_, *v*_*j*_)_ is a learnable scalar indexed by *ϕ*(*v*_*i*_, *v*_*j*_) and is shared among all layers. The spatial encoding is generalized into the following form:

$$\begin{array}{c} \Psi_{ij}^{attn}=\frac{\left(h_{i}W_{Q}\right){\left(h_{j}W_{K}\right)}^{⊤}}{\sqrt{d}}+b_{\phi (v_{i},v_{j})}^{\prime }, \end{array}$$ (20)

$$\begin{array}{c} b_{\phi (v_{i},v_{j})}^{\prime }=\frac{1}{1+e^{-(-k\phi \left(v_{i},v_{j}\right)-b)}}, \end{array}$$ (21)

where *ϕ*(*v*_*i*_, *v*_*j*_) describes the connectivity between nodes in the graph and sets the output value as the shortest path between node *v*_*i*_ and node *v*_*j*_. If the two nodes are not connected, the output value of *ϕ* is set to −1. Both *k* and *b* are learnable parameters and are shared among all layers. Our purpose is to set $b_{\phi (v_{i},v_{j})}^{\prime }$ to a decreasing function of *ϕ*, so that the model pays more attention to the nodes near each node and less attention to distant nodes. Regarding the spread of infectious diseases between cities, the closer the distance between two cities, the greater the probability of spread; that is, the higher the correlation between the epidemics in cities [1, 2].

**Edge encoding:** Edge features are important for graph representation. Encoding edge features into the network along with node features can significantly improve the model’s representation and performance. Especially in graphs containing complex relationships, edge features provide additional information that helps in more accurately modeling the correlations between nodes. The Graphormer uses an edge encoding that, for each node pair (*v*_*i*_, *v*_*j*_), finds the shortest path *SP*_*ij*_ = (*e*_1_, *e*_2_, …, *e*_*N*_), the average of the dot product of edge features and learnable embedding is computed and introduced as a bias term into the attention module. From this, the element $\Psi_{ij}^{attn}$ of the *i*^*th*^ row and *j*^*th*^ column of the matrix Ψ^*attn*^ is further updated to:

$$\begin{array}{c} \Psi_{ij}^{attn}=\frac{\left(h_{i}W_{Q}\right){\left(h_{j}W_{K}\right)}^{⊤}}{\sqrt{d}}+b_{\phi (v_{i},v_{j})}+\varepsilon_{ij},\;\;where\;\;\varepsilon_{ij}=\frac{1}{N}\sum_{n=1}^{N}x_{e_{n}}{\left(w_{n}^{E}\right)}^{⊤}, \end{array}$$ (22)

where $x_{e_{n}}$ is the feature of the *n*^*th*^ edge *e*_*n*_ in *SP*_*ij*_, $w_{n}^{E}\in R^{d_{E}}$ is the *n*^*th*^ weight embedding, and *d*_*E*_ is the dimensionality of the edge feature. In our model, the edge features are introduced directly into the attention module via bias terms, which leads to a simple generalization of the following form:

$$\begin{array}{c} \Psi_{ij}^{attn}=\frac{\left(h_{i}W_{Q}\right){\left(h_{j}W_{K}\right)}^{⊤}}{\sqrt{d}}+b_{\phi (v_{i},v_{j})}^{\prime }+e(v_{i},v_{j}), \end{array}$$ (23)

where *e*(*v*_*i*_, *v*_*j*_) denotes the edge features pointing from node *v*_*i*_ to node *v*_*j*_, i.e., it portrays the effect of mobility between nodes on the spread of the epidemic between two nodes.

### Temporal Convolution Layer

Dilated causal convolution [35] is commonly used to deal time series data. Unlike traditional convolution, dilated causal convolution has the property of dilation, whereby the receptive field is increased by skipping a certain number of step values instead of scanning them one by one as in normal convolution, and by increasing the depth of the convolution layers, which allows the network to capture a wider range of input information, thus achieving a larger receptive field. “Causal” means that the convolution operation uses only information from before the current time step and not from the future, maintaining temporal causality. Assuming that **x** ∈ *R*^*T*^ is a one-dimensional sequence and **f** ∈ *R*^*K*^ is a convolution kernel, the dilated causal convolution operation between **x** and **f** at time step *t* is denoted as:

$$\begin{array}{c} x⋆f\left(t\right)=\sum_{s=0}^{K-1}f\left(s\right)x\left(t-o\times s\right), \end{array}$$ (24)

where o is the dilation factor. By increasing the dilation factor, the sensory field of the model can be increased, and longer sequence information can be captured efficiently without increasing the number of layers. This is useful for dealing with time-series data with long-term dependencies and can improve the performance of the model while maintaining computational efficiency.

#### Gated TCN

We use Gated TCN [36] as the Temporal Convolution Layer to capture temporal trends in the node data. The introduction of a gating mechanism helps the network learn and control the flow of information in the time series data more efficiently. It takes the following form:

$$\begin{array}{c} H_{T}^{l}=g\left(\theta_{1}⋆H^{l}+b_{1}\right)⊙\sigma \left(\theta_{1}⋆H^{l}+b_{2}\right), \end{array}$$ (25)

where *H*^*l*^ is the input to the *l*th layer of the Gated TCN and $H_{T}^{l}$ is the output of the *l*th layer of the Gated TCN, *θ*_1_ and *θ*_2_ are the temporal convolution kernels, *b*_1_ and *b*_2_ are the biases, *g*(⋅) is the *tanh* activation function applied to the output, *σ*(⋅) is the *sigmoid* activation function that forms the gate, and ⊙ is the corresponding elemental product.

### Graph and Contact Prediction Layer

We use the same method to predict intra-city travel intensity (ICTI) and dynamic mobility data for the next *T*_*out*_ days. Unlike the general methods, the two modules are made to share a learnable graph. By sharing the learnable graph, the predicted data are viewed as a weighted average of the previous *T*_*in*_ days. In addition, the parameters of the graph and the contact rate prediction layer can be updated by gradients in the spatio-temporal layer and in the dynamics model, making the learned results more realistic.

**Fig 5:**
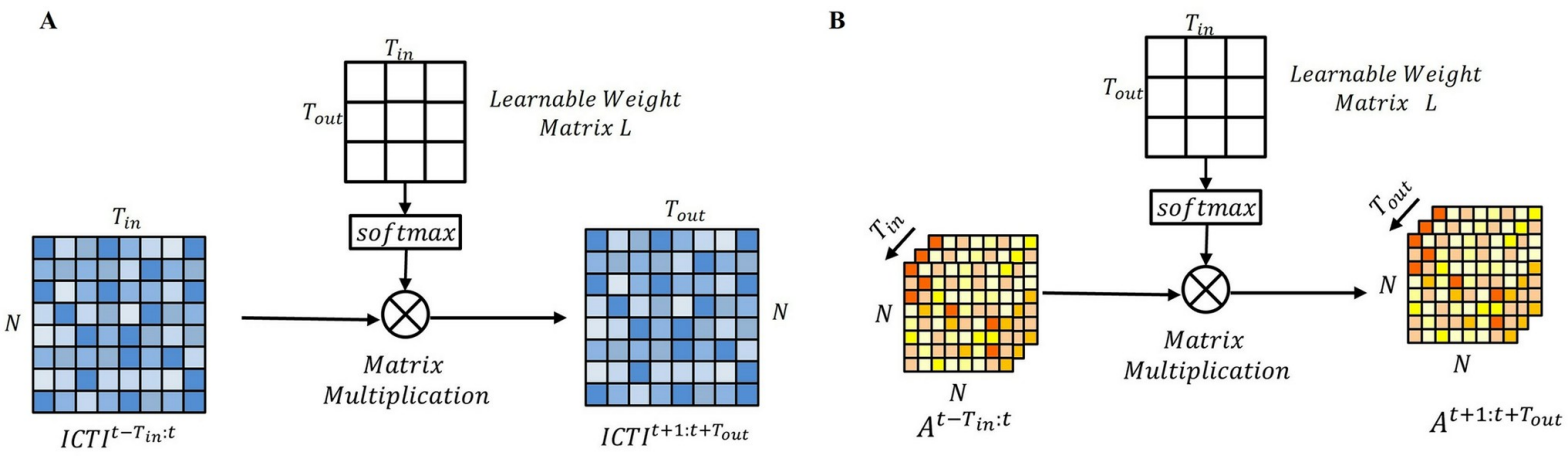
Framework of ICTI and dynamic flow prediction. A: Intra-city travel intensity prediction. B: Dynamic flow (weighted adjacency matrix *A*) prediction. Here softmax represents the softmax activation function.

As shown in Fig 5, there are two types of input data. The first type is shown in Fig 5A, which uses the historical *T*_*in*_ days of intra-city travel intensity $ICTI^{t-T_{in}:t}\in R^{N\times T_{in}}$. Initialize a learnable time weight matrix $L\in R^{T_{out}\times T_{in}}$, and normalize it to $\tilde{L}$ by rows using the *softmax* function. The normalized time weight matrix $\tilde{L}$ can map the historical *T*_*in*_ day intra-city travel intensity data $ICTI^{t-T_{in}:t}$ to the future *T*_*out*_ day in-town travel intensity $ICTI^{t+1:t+T_{out}}\in R^{N\times T_{out}}$, calculated as follows:

$$\begin{array}{c} \tilde{L}=soft\underset{:,j}{\text{max}}\left(L\right), \end{array}$$ (26)

$$\begin{array}{c} ICTI^{t+1:t+T_{out}}=\tilde{L}\;ICTI^{t-T_{in}:t}. \end{array}$$ (27)

The second type is shown in Fig 5B, which uses the weighted adjacency matrix (dynamic mobility data) $A^{t-T_{in}:t}\in R^{N\times N\times T_{in}}$ for historical *T*_*in*_ days, and uses the same learnable temporal weight matrix and normalized matrix $\tilde{L}$. The normalized temporal weight matrix $\tilde{L}$ can be mapped from the weighted adjacency matrix of the historical *T*_*in*_ days to the weighted adjacency matrix of the future *T*_*out*_ days $A^{t+1:t+T_{out}}\in R^{N\times N\times T_{out}}$, calculated as follows:

$$\begin{array}{c} A^{t+1:t+T_{out}}=\tilde{L}A^{t-T_{in}:t}. \end{array}$$ (28)

ICTI is a useful indicator of the intensity of human activity and is used as a measure of the effectiveness of interventions. However, ICTI only indicates activity intensity, and more detailed data such as geographic location are not fully utilized to directly infer changes in contact rate. It is often assumed that when people travel less, there is a corresponding reduction in contact rate. However, studies have shown that similar levels of contact are observed at high and low levels of traveling [37, 38]. Inspired by [39], a functional relationship between the intensity of ICTI and the average contact rate was constructed for contact rate prediction:

$$\begin{array}{c} c_{i}\left(t\right)=\text{ln}\left({\left[PD_{i}^{t}\times ICTI_{i}^{t}\right]}^{C_{F_{i}}^{t}+a}\right)+b_{c}, \end{array}$$ (29)

where $PD_{i}^{t}$ is the population density of patch *i*, $C_{F_{i}}^{t}\in C_{F}^{t}$ is a factor affecting the contact rate of patch *i*, *a* is a hyper-parameter and *b*_*c*_ is noise.

## Results

### Data

We obtained the daily number of new COVID-19 confirmed cases, the daily number of new discharges, and the cumulative number of confirmed cases for a total of 334 days from 1 January 2022 to 30 November 2022, for each province from the provincial health commissions under the National Health Commission of China [40], respectively. Changes in movement data for each provincial area was generated from the streaming data (activity trajectories) reported by the Beijing Municipal Commission of Health [41] from the 139th confirmed case to the 2,369th confirmed case, which represents the change in the extent of human movement compared to the baseline period. Natural birth rate, natural death rate, resident population and area were obtained from the seventh population census of the National Bureau of Statistics of China [42]. The in-migration (out-migration) size index, in-migration (out-migration) ratio, and intra-city travel intensity of all prefecture-level cities from 1 January 2022 to 30 November 2022 were obtained from Baidu Migration [43], and the number of people who moved in and out of the city per day was inferred and performed based on Baidu’s migration size index by the method proposed by [44]. Since Baidu Migration does not provide the intra-provincial travel intensity of each province, we approximate the intra-provincial travel intensity of each province by averaging the intra-city travel intensity of all prefecture-level cities in each province to obtain the intra-provincial travel intensity for each province.

### Model calibration

**Fig 6:**
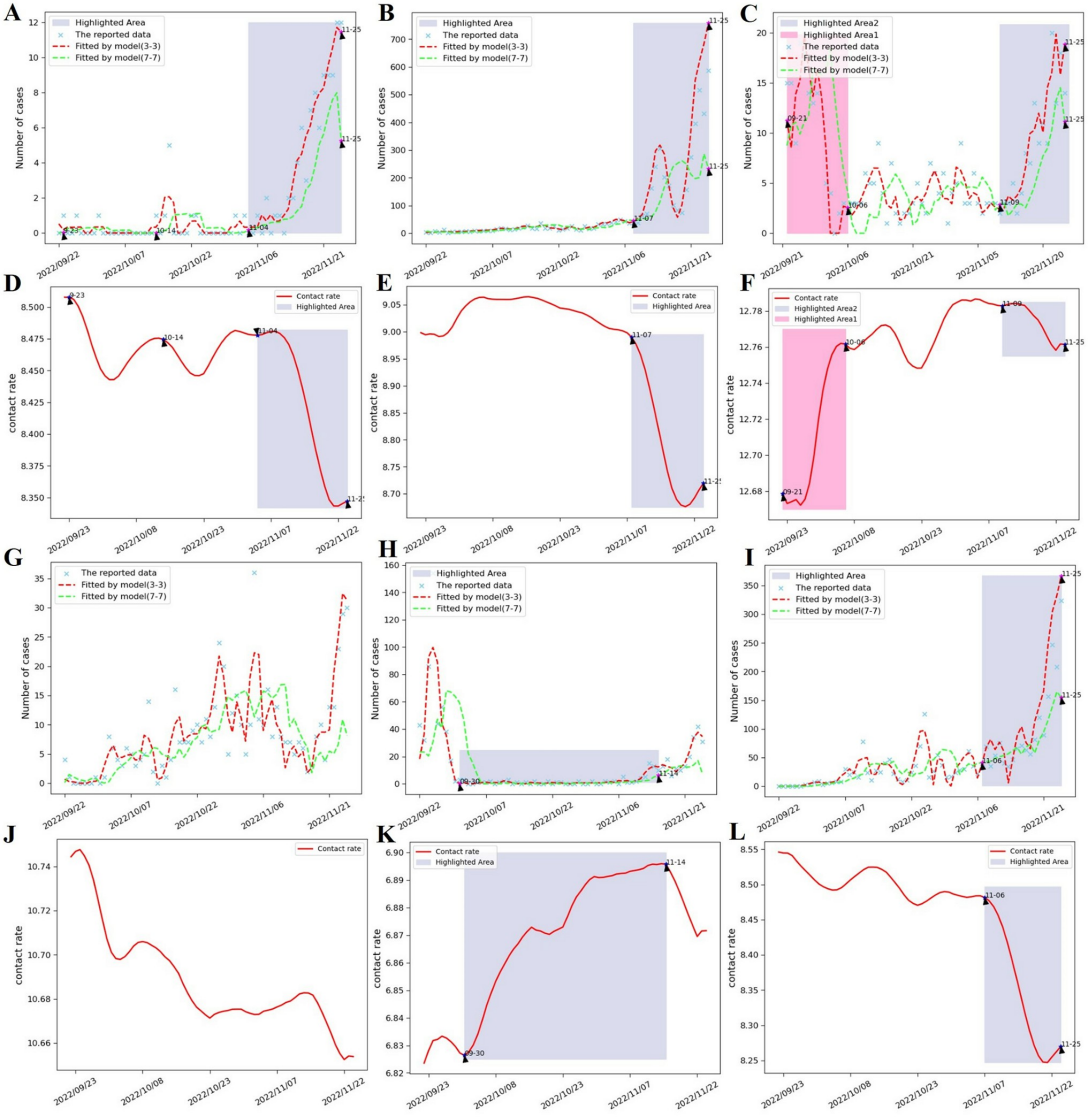
Fitting of daily new case data and time-dependent contact rate inference in Hubei, Beijing, Shanghai, Hunan, Guizhou, and Shanxi provinces based on M-Graphormer. Fig 6A-6C and 6G-6I represent the fitting results of daily new cases in Hubei, Beijing, Shanghai, Hunan, Guizhou, and Shanxi respectively, where the cyan ‘×’ represents the real reported data, and the red (the green) dotted line indicates that the model inputs 3 days (7 days) of historical data to predict daily new cases in the next 3 days (days). Fig 6D-6F and 6J-6L represent the time-dependent contact rate inferences for Hubei, Beijing, Shanghai, Hunan, Guizhou, and Shanxi, respectively. The highlighted areas in the two types of graphs correspond to the specific periods that we have drawn.

**Fig 7:**
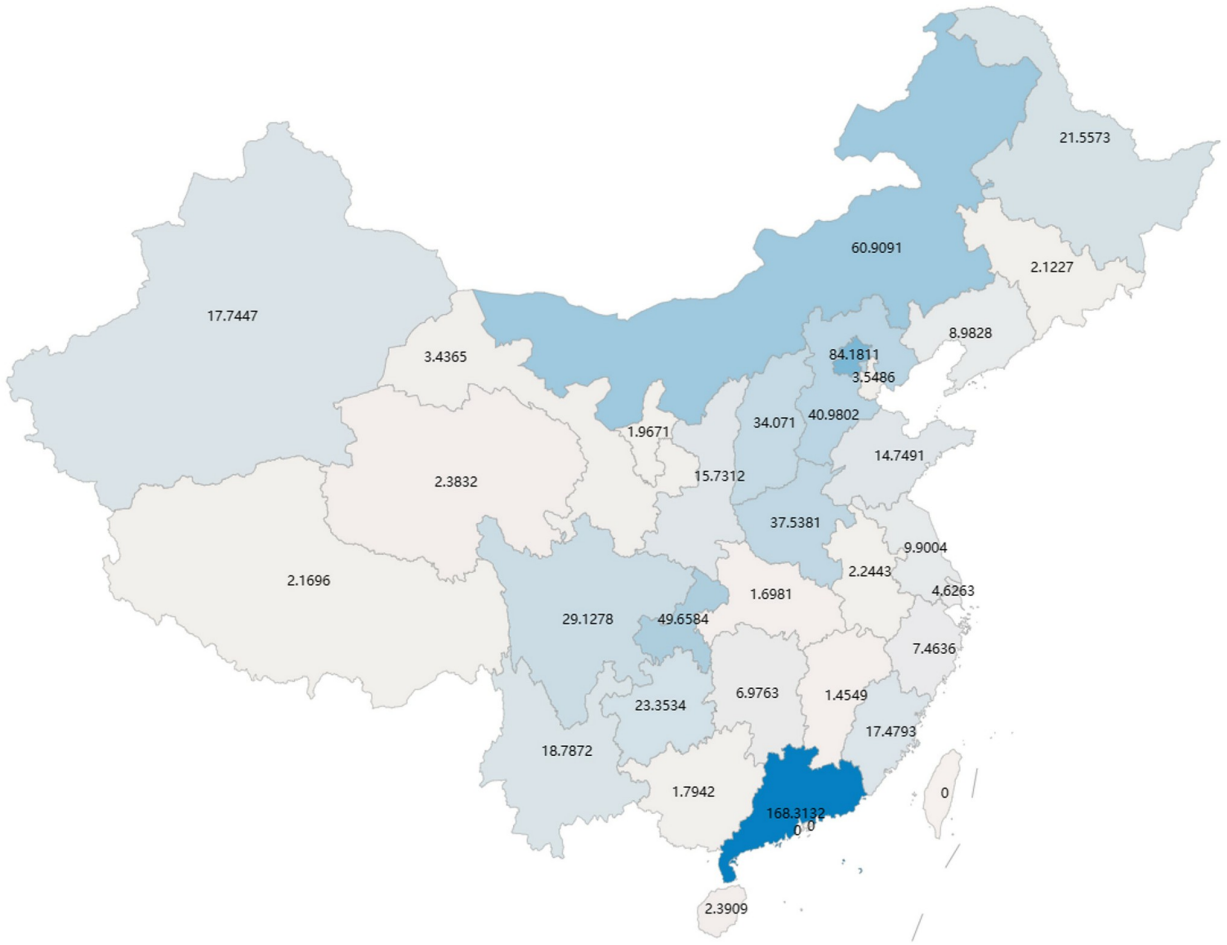
Map of cumulative daily root-mean-square errors for each province in China in the test set. The map was drawn in python using the pyecharts package (https://github.com/pyecharts).

**Fig 8:**
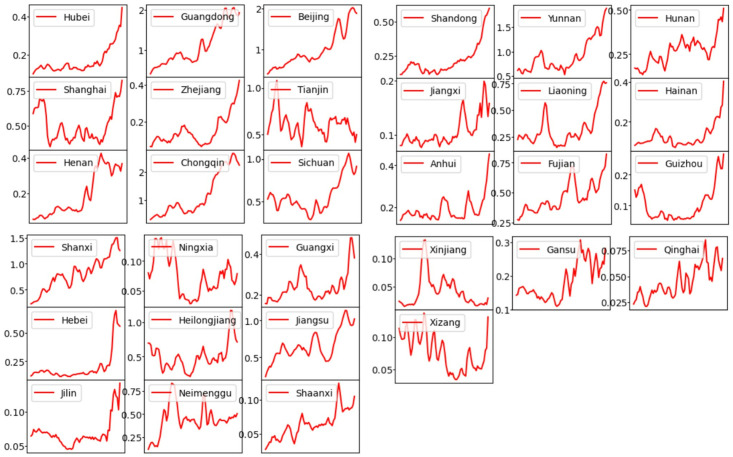
Effective reproduction number curves for each province in China estimated by Eq 14. The x-axis represents the dates, from September 23, 2022 to December 1, 2022.

In this study, simultaneous multi-region COVID-19 prediction of the number of new cases per day and simultaneous inference of high-dimensional epidemiological parameters were performed using M-Graphormer. All the data were divided into training, validation and test sets in the ratio of 3:1:1, and all the prediction results were performed in the test set so that the model performance could be better tested. Assuming a recovery rate constant *γ* = 0.125, and from [45], the death rate due to disease λ = 0.00008 is obtained. We show the prediction results for some regions, such as Hubei, Beijing, Shanghai, Hunan, Guizhou, and Shanxi, respectively, in Fig 6, where the daily new prediction results contain two curves for predicting the number of daily new cases in the next 3 days (7 days) using 3 days’ (7 days’) historical data. Fig 7 is a plot of the cumulative root-mean-square error (RMSE) between the predicted number of new cases per day and the actual reported data for each province/region from 23 September 2022 to 30 November 2022, which is set to 0 because the data used excludes Hong Kong, Macao, and Taiwan Provinces. Fig 8 shows the effective regeneration curves of each provincial region from September 23, 2022 to December 1, 2022.

From Fig 6A–6C, 6G–6I and 7, it can be concluded that the M-Graphormer can obtain predictions for multiple regions simultaneously under conditions of poor data quality and can fit the multi-wave epidemic data well. As can be derived from Fig 6D–6F and 6J–6L, the model can also simultaneously infer contact rate over time under different interventions implemented in different regions. We find that the number of new cases per day and contact rate over time predicted by the model have the same epidemic progression, which can be explained by the fact that China adopts a dynamic zeroing policy, where interventions are stepped up as outbreaks are detected. And the results predicted for specific periods are specifically labeled in Fig 6. For example, in Fig 6B, the outbreak begins in Beijing on 7 November 2022, interventions are intensified, and the corresponding contact rate curve in Fig 6E simultaneously falls off a cliff, consistent with the course of the outbreak in Fig 6B. In Fig 6H, Guizhou has a wave of outbreaks until 30 September 2022 and the outbreak almost disappears between 30 September 2022 and 14 November 2022, corresponding to the contact rate change curve in Fig 6K, where the contact rate is at a very small value on 30 September 2022 and the outbreak almost disappears between 30 September 2022 and 14 November 2022. During this period, the contact rate increases substantially, in line with the course of the epidemic.

All the results show that M-Graphormer can effectively deal with the dynamic graph structure due to human mobility and is able to predict multi-wave outbreaks in multiple regions using heterogeneous data from multiple sources, as well as accurately inferring changes in high-dimensional parameters such as contact rate. The model more accurately restores unobserved epidemic dynamics through limited, sparse and noise-affected data, and completely reconstructs the course of the epidemic. In addition, based on the predicted contact rate and the number of new cases per day, we find that the contact rate shows a decreasing trend in a given period (e.g., shown in the highlighted areas of Fig 6E, 6J and 6L), while the number of new cases per day shows an increasing trend (e.g., shown in the highlighted areas of Fig 6B, 6G and 6I). This is related to the rise in the intensity of interventions and the strengthening of localized blockades due to China’s dynamic clearance policy once an outbreak has occurred. In order to better quantify the evolution of the intervention, a specific rate function was chosen to describe the pattern of temporal evolution of contact rate in a given period.

### Ablation study

In order to demonstrate the effect of the different components of the M-Graphormer model, the ablation of the model was investigated in two separate prediction modes. We introduced the following three model variants: (1) w/o SIR: Remove the dynamics model completely; (2) w/o Eq (29): Remove Eq (29); (3) w/o Three encoding: Remove all three encoding designs from the Graphormer Layer. Two metrics are used to evaluate the performance: RMSE, MAE (Mean Absolute Error). To mitigate the effects of randomness, we performed five trials for each model and calculated the mean and 95% confidence interval of the results. The random seeds used were 0,1,2,3,4. The results are recorded in Table 1, where the column ‘Prediction modes’ indicates the number of new cases per day for the next 3 days(7 days) using 3 days(7 days) of historical data. From the results, we can make several observations. *Firstly*, the removal of the dynamical model produced a dramatic decrease in the performance of the model, which confirms that the dynamical model is crucial. *Second*, our full model obtained better performance in the experiments, which verifies that all three modules can significantly improve the performance of our model.

**Table 1:** Ablation study.

Prediction modes	Model	RMSE	MAE
3-3	w/o SIR	11642.0 ± 411.2	4108.9±243.8
	w/o Eq (29)	177.4±41.7	52.7±7.2
	w/o Three encoding	168.4±29.1	52.5±9.3
	M-Graphormer	**167.24±22.1**	**51.7±3.9**
7-7	w/o SIR	13082.8 ± 530.4	4854.8±446.7
	w/o Eq (29)	326.9±63.9	93.2±9.2
	w/o Three encoding	354.9±47.1	92.4±12.9
	M-Graphormer	**313.8±38.6**	**92.2±7.7**

### Interpretability analysis of time-dependent parameters

In order to further quantify the pattern of temporal evolution of contact rate under the implementation of different interventions in different regions, the three rate functions mentioned in [26] were introduced. The expressions for these three rate functions are given below:

$$\begin{array}{c} \left\{ \begin{array}{c} c^{1}\left(t\right)=\left(c_{01}-c_{b1}\right)e^{-r_{11}t}+c_{b1},\\ c^{2}\left(t\right)=\left(c_{02}-c_{b2}\right)e^{-{\left(r_{12}t\right)}^{2}}+c_{b2},\\ c^{3}\left(t\right)=c_{b3}{\left[1+({\left(\frac{c_{b3}}{c_{03}}\right)}^{-\eta }-1)e^{-r_{13}\eta t}\right]}^{\frac{1}{\eta }}. \end{array}\right. \end{array}$$ (30)

The main parameters in the Eq (30) are defined in Table 2. The contact rate series inferred in M-Graphormer are used as observations, and they are denoted as $\hat{c}\left(t\right)$. Since the rate functions in Eq (30) are all decreasing functions (i.e., quantifying increasing interventions), the highlighted area in Fig 6E indicates that the contact rate in Beijing is in a decreasing trend from 7 November 2022 to 17 November 2022, the highlighted area in Fig 6J indicates that the contact rate in Hunan is in a decreasing trend throughout the interval from 21 September 2022 to 25 November 2022, and the highlighted area in Fig 6L indicates that the contact rate in Shanxi was in a decreasing trend from 6 November 2022 to 17 November 2022, so these three regions and time periods were used as observations separately. Use Θ to denote the parameters to be estimated in the three rate functions, and use the least squares method as the method to estimate the parameters. It’s equivalent to solving the optimization problem of:

$$\begin{array}{c} \underset{\Theta }{\text{arg}\;\text{min}}\;L_{c^{i}}\left(\Theta \right)=\sum_{t=1}^{T}|c^{i}\left(t,\Theta \right)-\hat{c}\left(t\right){|}^{2},i=1,2,3. \end{array}$$ (31)

**Table 2:** Definition of commonly used parameters of the rate function.

Variables	Description
*c*_0*i*_ (*i* = 1, 2, 3)	Contact rate at the initial time
*c*_*bi*_ (*i* = 1, 2, 3)	Minimum contact rate under the current control strategies
*r*_1*i*_ (*i* = 1, 2, 3)	Exponential decreasing rate of contact rate
*η*	Interference constant

**Fig 9:**
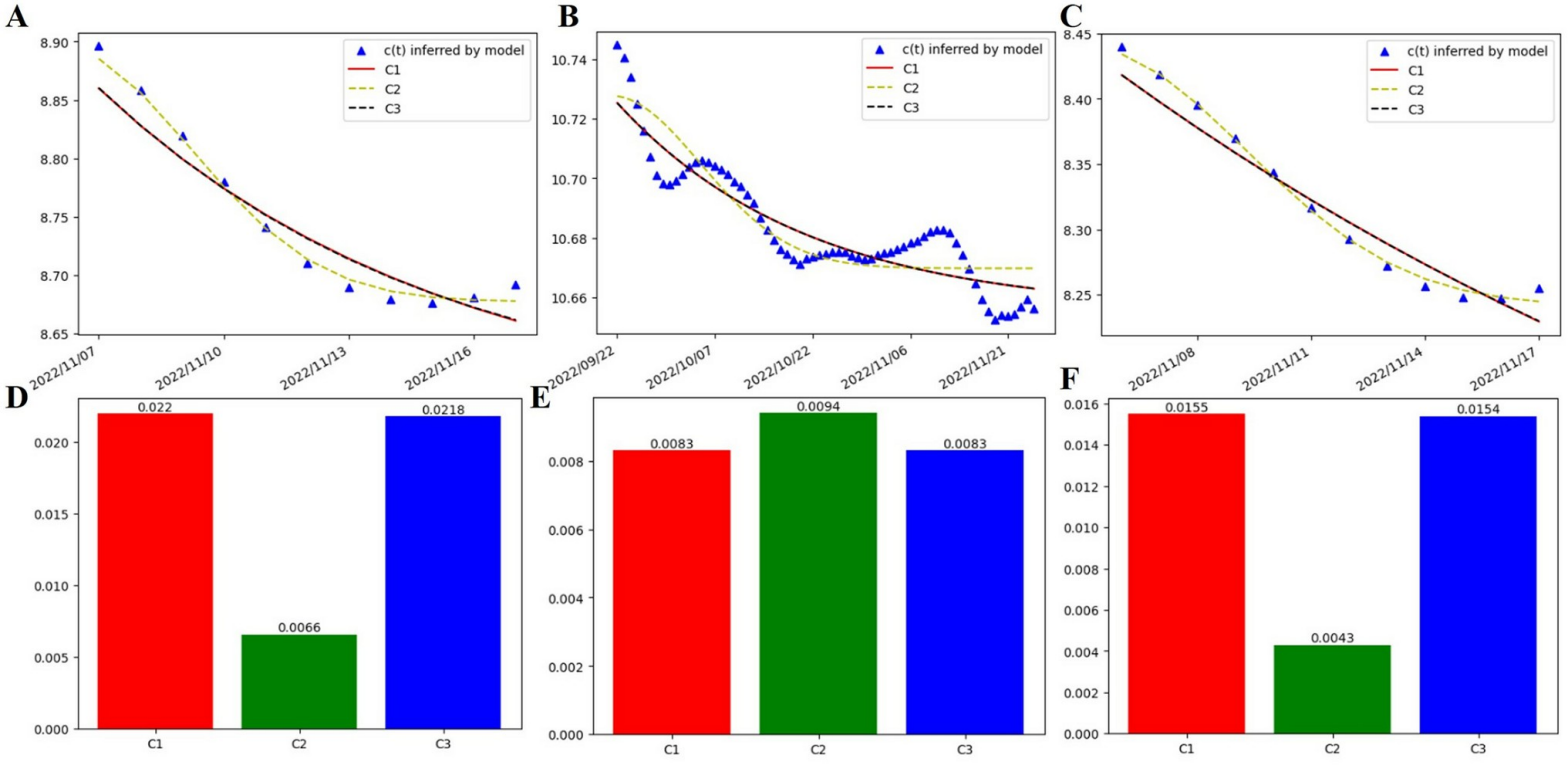
Contact rate inference, rate function fitting and root mean square error in Beijing, Hunan and Shanxi. A-C show the inference and fitting results of time-dependent contact rate in Beijing, Hunan and Shanxi, where the blue triangles represent the M-Graphormer inference results on the contact rate, and the dotted lines and realisations represent the rate function group (30) fitting results. D-F show the root mean square errors corresponding to the fitting results of the rate function group (30) in Beijing, Hunan and Shanxi.

The optimal parameters obtained by least squares for the three regions and periods are shown in Table 3, and the fitting results are presented in Fig 9A–9C. In order to select the most suitable function to describe the extrapolated results of the contact rate for a specific period, the root mean square error of the fitting results of the three rate functions were calculated separately for each region and are shown in Fig 9D–9F. It can be concluded that for Beijing, the function *c*^2^(*t*) can be chosen to describe the extrapolated results of the contact rate in that period. For Hunan, either *c*^1^(*t*) or *c*^3^(*t*) can be chosen to describe the extrapolated results of the contact rate in that period, and for Shanxi, the function *c*^2^(*t*) can be chosen to describe the extrapolated results of the contact rate in that period. The above results indicate that, in order to cope with the epidemic, the prevention and control models in different regions should be flexibly adjusted according to their specific conditions, and should not be directly copied from other regions. That is, the prevention and control measures should be tailored according to the actual situation in each region.

**Table 3:** Definition of commonly used parameters of the rate function.

Variables	Beijing	Hunan	Shanxi	Sources
*c*_0*i*_ (*i* = 1, 2, 3)	8.8959	10.7278	8.4397	M-Graphormer
*c*_*b*1_	8.5732	10.6550	7.8714	Estimated
*c*_*b*2_	8.6773	10.6698	8.2412	Estimated
*c*_*b*3_	8.5871	10.6555	7.9507	Estimated
*r*_11_	0.1183	0.0342	0.0385	Estimated
*r*_12_	0.2234	0.0513	0.1666	Estimated
*r*_13_	0.0173	0.0035	0.0068	Estimated
*η*	8	10	8	Assumed

### Arrival times: Predictions

Predicting the arrival time of infectious diseases is of great importance in the field of public health and epidemic prevention. It can provide early warning and response opportunities, optimize resource allocation, and develop effective prevention and control strategies in advance. This capability significantly enhances the response capacity of the public health system, mitigates the negative impacts of infectious diseases, and effectively protects public health. The time of arrival is defined as: Assuming that the disease originates in one city, how long does it take for it to appear in another city, i.e. how long does it take for the number of people infected by the disease to reach the threshold *κ* for the first time in a given city. Mathematically, the time for the disease to first appear in city *i* and reach city *j* is defined as:

$$\begin{array}{c} AT_{ij}=\text{inf}\left\{t\geq 0|I_{j}\left(t\right)=\kappa \right\}. \end{array}$$ (32)

The method proposed by [46] is used to estimate the arrival time of a nonlinear system in terms of a linearized arrival time approximation by linearizing it in the vicinity of the disease-free state. Then the arrival time is estimated as:

$$\begin{array}{c} AT_{ij}=\frac{p_{ij}}{\bar{\beta c}-\bar{\mu }-\lambda -\gamma -m}W\left(\frac{{\left(p_{ij}!\right)}^{\frac{1}{p_{ij}}}}{p_{ij}}\frac{\bar{\beta c}-\bar{\mu }-\lambda -\gamma -m}{m{\left(\rho_{p_{ij}}\right)}^{\frac{1}{p_{ij}}}}{\left(\frac{\kappa }{\chi_{0}}\right)}^{\frac{1}{p_{ij}}}\right), \end{array}$$ (33)

where $\bar{\beta c}=\frac{1}{n}\sum_{k}^{n}{\left(\beta c\right)}_{k}$, $\bar{\mu }=\frac{1}{n}\sum_{k}^{n}\mu_{k}$, *p*_*ij*_ denotes the shortest path (minimum number of nodes passed through) from node *i* to node *j*. $\rho_{p_{ij}}=\xi_{i}^{⊤}P^{p_{ij}}\xi_{j}$ describes the probability that an individual starting from node *i* will be located at node *j* after *p*_*ij*_ steps. *χ*_0_ is the number of infected individuals in node *i* at the initial moment, *ξ*_*i*_ is the standard Euclidean basis vector and *W*(⋅) is the Lambert-W function.

In the Eq (12), some of the parameters are allowed to vary with node, and the method in Eq (33) assumes that the parameters are uniform across all patches. Consider how the non-uniformity of these parameters affects the arrival time, i.e., whether this non-uniformity speeds up or slows down the arrival time compared to the average. For convenience, we call dynamical models with identical parameters homogeneous systems, and dynamical models in which some of the parameters vary with the nodes non-homogeneous systems. In non-uniform systems, Eq (33) is no longer effective in estimating the arrival time, and the alternating derivation in [47] is used to estimate the arrival time of the non-uniform system. In this paper, only node pairs with *p*_*ij*_ = 1 are used to compare arrival times with the uniform system, assuming that the disease first appears at node *i* and node *j* is connected to it. Let

$$\begin{array}{c} \Gamma_{i}=\bar{\beta c}+\omega_{i}^{\beta c}-\bar{\mu }+\omega_{i}^{\mu }-\lambda -\gamma , \end{array}$$ (34)

where $\omega_{i}^{\beta c}={\left(\beta c\right)}_{i}-\bar{\beta c}$ and $\omega_{i}^{\mu }=\mu_{i}-\bar{\mu }$, the dynamics of the number of infected at node *n* = *j* is approximated by:

$$\begin{array}{c} I_{j}\left(t\right)\approx mP_{ji}\chi_{0}e^{\Gamma_{j}t}\int_{0}^{t}e^{-\Gamma_{j}\tau }e^{\Gamma_{i}\tau }d\tau =mP_{ji}\chi_{0}e^{\Gamma_{j}t}{\left[\frac{e^{(\omega_{i}-\omega_{j})\tau }}{\omega_{i}-\omega_{j}}\right]}_{0}^{t}, \end{array}$$ (35)

where $\omega_{i}=\omega_{i}^{\beta c}+\omega_{i}^{\mu }$. Setting Eq (35) to the threshold *κ*, two estimates of arrival time depending on the size relation between *ω*_*i*_ and *ω*_*j*_ are obtained:

$$\begin{array}{c} \left\{ \begin{array}{c} AT_{ij}\approx -\frac{1}{\Gamma_{i}}\text{log}(\frac{1}{m}\frac{\kappa (\omega_{i}-\omega_{j})}{\chi_{0}P_{ji}}),\;\omega_{i}>\omega_{j},\\ AT_{ij}\approx -\frac{1}{\Gamma_{j}}\text{log}(\frac{1}{m}\frac{\kappa (\omega_{j}-\omega_{i})}{\chi_{0}P_{ji}}),\;\omega_{j}>\omega_{i}, \end{array}\right. \end{array}$$ (36)

when *ω*_*i*_ > *ω*_*j*_, the infection growth of node *j* is dominated by the migration of infections from node *i*. On the contrary, if *ω*_*i*_ < *ω*_*j*_, the local infection growth of node *j* is dominated by node *i*, which only needs to propagate a small number of initial infections to node *j*.

**Fig 10:**
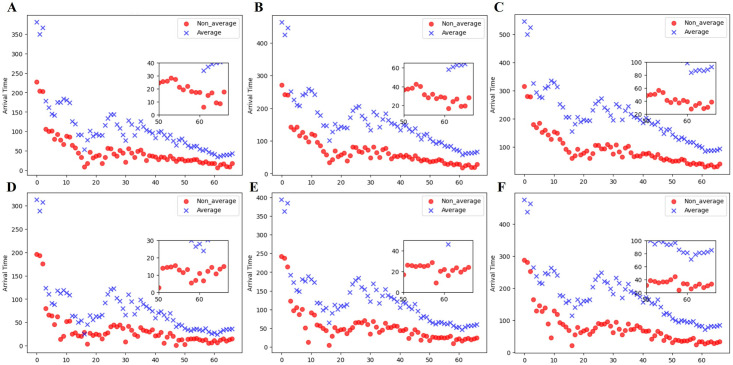
Arrival times of different thresholds *κ* for the two systems under different parameter groups. The abscissa represents the serial number of different parameter groups. A-C represent the arrival time from Shanxi to Guangdong at thresholds *κ* = 1, 10, 100, respectively. D-F represent the arrival time from Shanxi to Beijing at thresholds *κ* = 1, 10, 100, respectively. The blue ‘×’ represents the arrival time of the uniform system, and the red solid circle represents the arrival time of the non-uniformity system. The insets in A-F show the minimum arrival time. The x-axis denotes the different parameter group numbers and also the numbering of the date sequence in the test set.

We use the parameters inferred by M-Graphormer in the test set to predict arrival times. Since the inferred parameters are time series, all the parameters at each time step are treated as a parameter group. Shanxi is selected as the initial infected province under different parameter groups to predict the arrival time to Guangdong and Beijing, as shown in Fig 10. It shows from left to right the time when Shanxi propagates to Guangdong and Beijing under different parameter sets of the two systems when the threshold *κ* = 1, 10, 100, respectively. The results show that as the threshold value keeps increasing, the arrival time also keeps increasing, and the arrival time of the non-uniform system is faster than that of the uniform system, which is consistent with the findings of [47]. We believe that local infection rate are bound to vary due to a variety of factors, so it is reasonable to have considerable variations. For example, for popular provinces such as Beijing and Guangdong, where the population base is large and dense, it is reasonable to expect a rapid spread of the disease, which confirms the findings of [48] that pandemics may occur earlier in large cities than in smaller ones. In addition, the actual arrival times of several cities mentioned in [49] were used as the actual arrival times of the provinces, and various epidemiological parameters and daily additions from 10th January to 20th January 2020 were estimated using the previously trained model. Both Eqs (33) and (36) were used and the arrival times for both systems were successfully estimated as shown in Table 4.

**Table 4:** Prediction of the actual time of arrival for both systems.

Province	Actual arrival time (day)	uniform system	nonuniform system
		Predicted arrival time (day)	Predicted arrival time (day)
Beijing	<23	37	30
Shanghai	<23	39	29
Guangdong	25	29	19
Sichuan	26	33	27
Yunnan	29	41	35
Liaoning	>30	46	28

## Discussion and conclusion

During the COVID-19 pandemic, China’s dynamic zero policy, especially high-frequency large-scale nucleic acid screening and closed management, played an important role in controlling the spread of the epidemic. In order to accurately predict the spreading trend of the epidemic, we retrospectively simulated the course of the COVID-19 epidemic in 31 provincial areas of China using the M-Graphormer and inferred high-dimensional epidemiological parameters.

M-Graphormer not only accurately predicts daily new cases and the course of the epidemic, as in Fig 6A–6C and 6G–6I, but also infers high-dimensional epidemiological parameters, as in Fig 6D–6F and 6J–6L. The results show that the use of different interventions in different regions produces different effects, and reveal that predefined functions may not accurately describe the actual contact rate. The highlighted areas in the corresponding contact rate change curves and new cases per day curves for each region in Fig 6 are found to depict the same epidemic progression, i.e., a gradual increase in new cases per day and a gradual decrease in the contact rate, as shown in Fig 6A, 6B, 6D and 6E, etc., which is consistent with the results of the previous studies [50, 51]. We introduced three rate functions from [26] and chose the appropriate function to describe the extrapolated results of the contact rate for Beijing, Hunan and Shanxi for a specific period of time as shown in Fig 9. We predicted the arrival time using two systems of dynamic model, taking Shanxi spreading to Guangdong and Beijing as an example, and used the actual epidemic data to prove the research conclusion in [47] that the arrival time of the non-uniform system is faster than that of the uniform system. At the same time, using the trained model, we successfully predicted the actual arrival time of the epidemic spreading from Wuhan to other provinces at the beginning of the epidemic.

In this study, a hybrid framework, Metapopulation Graph Transformer Neural Network (M-Graphormer), was constructed by incorporating the Graphormer and graph learning mechanisms into the metapopulation SIR model. It enables the neural network to follow the rules of infectious disease dynamics during the learning process, integrating real-time data and complex infectious disease transmission patterns. It also extends the three graph structure encoding included in the Graphormer so that it can be more applicable to epidemiological scenarios. The hybrid framework avoids the loss of certain hidden spatial dependencies in dynamic graph data, and is able to learn high-dimensional epidemiological parameters and simultaneously predict the spreading state of multi-region epidemics in an end-to-end manner using heterogeneous data from multiple sources. At the same time, the model can restore the unobserved epidemic dynamics more accurately and reconstruct the epidemic development process completely with limited, sparse and noise-affected data. In addition, the method can be easily extended to other more complex models of network infectious disease dynamics, allowing a more comprehensive understanding of the impact of different factors on the spread of infectious diseases, which is important for the study of infectious diseases and the development of prevention and control measures.

We believe that the vast majority of infectious diseases spread through human mobility can be effectively applied to this new method. For example, diseases with obvious geographical and temporal transmission characteristics, such as influenza, can be modeled and predicted with the help of this model. However, it is worth noting that since this method is based on deep learning and is a data-driven model, its performance depends on a large amount of high-quality training data. In order for the model to fully learn effective transmission laws and obtain good prediction results, a sufficient sample size is usually required. If the amount of training data is small, the model may not be able to effectively capture complex flow patterns, resulting in insufficient prediction performance. In addition, due to the particularity of human mobility data, many real-world data (such as population mobility, migration trajectories, etc.) may be difficult to obtain or incomplete, which also brings certain challenges to the application of the model. Therefore, in practical applications, the quality, availability and representativeness of the data will be key factors affecting the effectiveness of the model.

Our study has some limitations. The dynamic model only considered the migration term and did not take into account the effect of short-staying people, such as commuters or travelers, and ignored the effect of measures such as vaccination and quarantine on the spread of infectious diseases. When setting a mobility threshold of 100 for constructing a weighted adjacency matrix to obtain a sparse graph structure, we did not consider the potential impact of different thresholds on the final prediction results, and experiments and evaluations may be needed to determine the mobility threshold that best suits the task requirements. After constructing the sparse graph structure, comparative experiments should be conducted to evaluate the effects of different thresholds on the final prediction results. Because of the lack of fine-grained movement change data, we generated movement change data for all provinces in the country using flow-regulation data from a particular region, which may not accurately reflect the differences and characteristics between provinces due to differences in their level of economic development and cultural background, and may be lacking in the characteristics of the nodes used, such as differences in healthcare resources and changes in GDP in different regions. In predicting the actual arrival times in other provinces at the beginning of the epidemic, using a model trained from the 2022 epidemic data to estimate the epidemiological parameters at the beginning of the epidemic may lead to inaccuracies because the early stages of the epidemic are very different from the later stages of transmission. We leave this for future work.
